# Tumor microbiome-transcriptome crosstalk identifies *Prevotella* as an immunotherapeutic predictor in NSCLC

**DOI:** 10.7150/thno.126091

**Published:** 2026-01-01

**Authors:** Na Wang, Lifang Ma, Yugang Huang, Xionghui Zhou, Yuan Rong, Fei Long, Wanbo Qiu, Si Wu, Yue Hu, Xin He, Jiurong He, Sufang Tian, Weidong Hu, Chunhui Yuan, Fubing Wang

**Affiliations:** 1 Department of Pathology, Renmin Hospital of Wuhan University, Wuhan 430060, China.; 2 Department of Clinical Laboratory Medicine, Shanghai Chest Hospital, Shanghai Jiao Tong University School of Medicine, Shanghai 200030, China.; 3 Department of Laboratory Medicine, Zhongnan Hospital of Wuhan University, Wuhan 430071, China.; 4 College of Informatics, Huazhong Agricultural University, Wuhan 430070, China.; 5 Department of Pathology, Zhongnan Hospital of Wuhan University, Wuhan 430071, China.; 6 Department of Thoracic Surgery, Zhongnan Hospital of Wuhan University, Wuhan 430071, China.; 7 Department of Laboratory Medicine, Wuhan Children's Hospital (Wuhan Maternal and Child Healthcare Hospital), Tongji Medical College, Huazhong University of Science & Technology, Wuhan 430016, China.; 8 Department of Clinical Laboratory, Renmin Hospital of Wuhan University, Wuhan 430060, China.; 9 Center for Single-Cell Omics and Tumor Liquid Biopsy, Zhongnan Hospital of Wuhan University, Wuhan 430071, China.; 10 Wuhan Research Center for Infectious Diseases and Cancer, Chinese Academy of Medical Sciences, Wuhan 430071, China.

**Keywords:** NSCLC, microbiome, transcriptome, *Prevotella*, immunotherapy response

## Abstract

**Background:** The tumor-resident microbiome plays a pivotal role in shaping the tumor immune microenvironment; however, its relationship with the host transcriptome and the response to immune checkpoint inhibitors (ICIs) remains largely uncharacterized in non-small cell lung cancer (NSCLC). This study aimed to elucidate the relationship between tissue-resident microbiota, host transcriptomic alterations, and immunotherapy response in NSCLC.

**Methods:** Paired tumor (T) and paracancerous tissue (PT) samples from patients with NSCLC were analyzed using 2bRAD-M and bulk RNA sequencing to generate comprehensive microbiome and transcriptome profiles. The conditional mutual information algorithm was employed to systematically investigate intratumoral microbe-host interactions. Associations between key microbes and patient prognosis, ICI response, and response to epidermal growth factor receptor (*EGFR*)-targeted therapy were assessed across four independent local clinical cohorts.

**Results:** Higher microbial richness, α-diversity, and β-diversity were observed in PT samples than in T samples. Specifically, PT-resident *Bradyrhizobium* and* Prevotella* were identified as key bacterial taxa significantly associated with immune cell populations, including CD8^+^ T cells, natural killer cells, and activated dendritic cells. Among these, PT-resident *Prevotella*, but not *Bradyrhizobium*, was independently associated with improved prognosis of patients with NSCLC and ICI response in both local clinical sets and public datasets. Furthermore, a combined diagnostic model integrating PT-resident *Prevotella* abundance with routine clinical blood indicators demonstrated markedly superior predictive performance for ICI response compared with the conventional biomarker PD-L1. By contrast, PT-resident* Prevotella* exhibited no association with treatment response in the *EGFR*-targeted therapy cohort.

**Conclusion:** PT-resident *Prevotella* is strongly associated with the prognosis and ICI response in patients with NSCLC. Moreover, integration of PT-resident *Prevotella* with routine clinical blood indicators holds promise as a potential auxiliary diagnostic tool to facilitate personalized immunotherapy in NSCLC.

## Introduction

Restoring antitumor immunity through immunotherapy has become a cornerstone of modern cancer treatment and has demonstrated remarkable efficacy in a subset of patients 1-4. Immune checkpoint inhibitors (ICIs) are now incorporated into first-line therapy for non-small cell lung cancer (NSCLC), and recent clinical trials (NCT03191786) have reported an increase in the 2-year survival rate for patients with advanced-stage disease from 12% to 24% 5. Despite their ability to alleviate immune suppression and reshape the tumor immune microenvironment, ICIs provide clinical benefit to approximately 40% of patients with NSCLC due to substantial interindividual variability in treatment response 6. Therefore, accurately identifying patients most likely to respond to immunotherapy is essential for advancing personalized treatment strategies in NSCLC.

Currently used clinical biomarkers for predicting ICI efficacy, such as programmed death-ligand 1 (PD-L1) expression, tumor mutational burden, and various gene expression signatures, offer limited predictive power, with area under the curve (AUC) values typically ranging from only 0.6 to 0.75 7. These biomarkers primarily reflect intrinsic tumor features, whereas response to immunotherapy depends on the dynamic and complex interactions between the tumor and the host 8. In recent years, several newly identified ICI-related biomarkers, such as CD8⁺ tumor-infiltrating lymphocytes 9 and T cell-inflamed gene expression profiles associated with antigen presentation, chemokine expression, cytolytic activity, and adaptive immune resistance 10, are all derived from intrinsic tumor features and demonstrate superior predictive performance compared with traditional biomarkers such as PD-L1. Therefore, efforts to identify novel immunotherapeutic biomarkers should focus on the immune system and its closely interacting components, such as tumor-associated microbiota, which predominantly reside within immune cells and form symbiotic relationships with the tumor immune microenvironment 11-13.

The tumor-resident microbiome, an emerging and integral component of the tumor microenvironment, plays a critical role in modulating host immune responses 11. In studies investigating the impact of the microbiome on tumor immunotherapy, early research has largely focused on the gut microbiome. For instance, the gut microbiota has been shown to critically modulate the efficacy of ICI therapy by shaping tumor-infiltrating immune cells and influencing macrophage polarization, thereby enhancing antitumor responses 14. In addition, microbiota-derived signals can reprogram mononuclear phagocytes within the tumor microenvironment toward immunostimulatory phenotypes, activating type I interferon-natural killer cell-dendritic cell signaling and improving the effectiveness of immune checkpoint blockade 15. With the development of microbiome sequencing technologies, it has become possible to accurately profile microbial communities within tumor tissues, providing novel insights into host-microbe interactions in the tumor microenvironment. Consequently, recent years have witnessed a growing body of evidence highlighting the presence of lung-resident microbes and their potential functional roles in pulmonary diseases 13, 16, 17. High intratumoral microbial diversity in NSCLC has been associated with improved patient survival 13. A more in-depth analysis further revealed that NSCLC tumors enriched with intratumoral microbes exhibit high expression of genes associated with favorable responses to ICIs, including *GZMB2*, *CCL20*, *CXCR2P124*, *CXCL1312*, and *IL12RB225*, suggesting that intratumoral microbes may enhance ICI efficacy by promoting an inflammatory tumor microenvironment 16. Although the overall abundance and diversity of intratumoral microbes are relatively limited, they can modulate the tumor immune status by activating innate immunity and regulating immune cell function during tumor immunoediting 18, 19. This unique characteristic offers strong theoretical and translational significance, given its potential to reflect the efficacy of immunotherapy. As open organs, the lungs harbor a particularly complex tumor immune microenvironment owing to the abundant infiltration of microorganisms 20. However, systematic studies integrating the intratumoral microbiome with the host transcriptome to identify predictive biomarkers of immunotherapy response in NSCLC are lacking.

By integrating microbiome and transcriptome data, we aimed to systematically elucidate the distribution patterns of tissue-resident microbes in patients with NSCLC. Our analysis revealed a significantly higher microbial abundance in paracancerous tissues (PT) than in tumor tissues (T), with no substantial differences observed between lung adenocarcinoma (LUAD) and lung squamous cell carcinoma (LUSC). Using the conditional mutual information (CMI) algorithm, we constructed a microbiota-host-gene interaction network that identified PT-resident *Prevotella* as closely associated with antitumor-related signaling pathways. Moreover, in both bulk and localized clinical cohorts, a higher abundance of PT-resident *Prevotella* was positively associated with response to immunotherapy and a favorable prognosis in NSCLC. Furthermore, a predictive model combining *Prevotella* abundance with routine blood test indicators demonstrated robust performance in predicting immunotherapy outcomes, providing a novel companion diagnostic approach to support personalized immunotherapeutic strategies in NSCLC.

## Materials and Methods

### Sample collection

A total of 20 paired fresh NSCLC tumor and adjacent normal tissue samples for sequencing were obtained from Zhongnan Hospital of Wuhan University. Additionally, tumor and adjacent normal tissue samples were collected from 94 NSCLC patients (**Table S1**) who received anti-PD-L1 immunotherapy at Zhongnan Hospital of Wuhan University (14 adult patients) and Shanghai Chest Hospital (80 adult patients), and from 52 NSCLC adult patients (**Table S2**) who underwent *EGFR*-targeted therapy at Taihe Hospital. Adjacent normal tissue samples were collected from regions located more than 5 cm away from the tumor margin. All samples were histologically verified by experienced pathologists through H&E staining to confirm the absence of tumor infiltration or airway contamination. The fresh tissue samples were rapidly frozen in liquid nitrogen within 30 min of the surgical resection. All instruments and materials in contact with the lung tissues were sterilised. Following the application of quality control exclusions, the final sequencing analysis was conducted on 17 samples of adjacent tissue and 18 samples of tumor tissue. The clinical data were collated by the attending physicians from the patients' clinical charts and hospital discharge records.

Immunotherapy and targeted therapy responses were evaluated radiologically every six weeks based on the Response Evaluation Criteria in Solid Tumors (RECIST) version 1.1. Patients achieving a complete response (CR), partial response (PR), or stable disease (SD) lasting ≥ 6 months were classified as having a clinical benefit response (CBR). In contrast, those with SD lasting < 6 months or progressive disease (PD) were categorized as having no clinical benefit (NCB).

### Tissue microarray

A LUAD tissue microarray (product No. HLugA180Su12; containing 90 paired tumor and adjacent normal tissues) and a LUSC tissue microarray (product No. HLug-Squl50Sur-02; containing 75 paired tumor and adjacent normal tissues) were purchased from Shanghai Outdo Biotech.

### 2bRAD sequencing for microbiome (2bRAD-M)

The 2b-RAD-M technology 21 is a qualitative and relative quantitative analysis of microorganisms that employs unique tags obtained through the enzymatic cleavage of microbial genomes by type IIB restriction enzymes. A database containing unique tags of each microorganism (2b-Tag-DB) was employed for qualitative analysis, whereby all microbial species that had unique tags were screened. The 2b-Tag-DB was then established again for the quantitative microorganisms, and a relative quantitative analysis was conducted. This entailed screening the microbial species obtained in the previous step and estimating their abundance according to the distribution of unique tags.

### Microbial diversity analysis and identification of differential taxa

The alpha diversity was calculated using the Chao1, Shannon and Simpson indices with the "vegan" package, and visualized as box plots 22. Beta diversity was assessed using Bray-Curtis, binary Jaccard and Euclidean distances, estimated by the "vegan" package and displayed as principal coordinate analysis (PCoA) scatter plots. Differential taxa between groups were identified using linear discriminant analysis (LDA) effect size (LEfSe), with an LDA score threshold of 4.0 23.

### Bacterial culture

A total of five pairs of T and PT samples obtained from patients with NSCLC were subjected to bacterial culture under both aerobic and anaerobic conditions. The bacterial culture procedure was performed following the method described by Huan Yu et al 24. Specifically, the obtained fresh tissue samples were immediately immersed in saline, with the entire sampling process conducted in accordance with strict aseptic conditions. Subsequently, in a sterile environment, tissue fragments were homogenised using a glass homogeniser in 1 mL of ice-cold PBS. Additionally, PBS was employed as a negative control, following the same workflow to ascertain the potential for environmental contamination. 100 μL of the aforementioned tissue homogenate was added to the BHI medium supplemented with 5% sheep blood. Pre-cultures was performed under aerobic or anaerobic conditions. After 24 h, the culture medium was inoculated onto Columbia agar medium with 5% sheep blood. The plates were incubated at 37 °C for 24 h under aerobic conditions or for 72 h under anaerobic conditions. Colonies were picked and identified using MALDI-TOF mass spectrometry (MS) systems (Autof MS1000).

### Immunohistochemical (IHC)

After dewaxed with xylene and hydrated with absolute ethanol, tissue sections were immersed in citric acid antigen retrieval buffer (pH 6.0). The sections were heated on medium heat until boiling for 8 min, taken off the heat for 8 min, followed by an additional 7 min on medium-low heat. The sections were then washed three times with PBS (pH 7.4) on a decolorization shaker, with each wash lasting 5 min. Subsequently, the sections were incubated in a 3% hydrogen peroxide solution at room temperature, protected from light, for 25 min, followed by three PBS washes. Next, the sections were blocked with 3% BSA at room temperature for 30 min. They were then incubated overnight at 4 °C with anti-lipopolysaccharide (LPS) antibody (HYCULT BIOTECH, HM6011) / lipoteichoic acid (LTA) antibody (HYCULT BIOTECH, HM2048). The following day, after three PBS washes, the sections were incubated at room temperature for 50 min with a horseradish peroxidase-conjugated goat anti-mouse secondary antibody (abcam, ab6789). Finally, the sections were developed using DAB (diaminobenzidine) and counterstained with hematoxylin. Positive expression was indicated by a brownish-yellow coloration.

### Fluorescence *in situ* hybridization (FISH)

FFPE tissue sections or tissue microarrays were deparaffinized and rehydrated. The sections were sequentially soaked in 100% xylene for 15 min twice, in 100% ethanol for 5 min twice, in 85% ethanol for 5 min, and finally in 75% ethanol for 5 min. Sections were washed in DEPC-treated water. Incubate the sections in boiling citrate-EDTA antigen retrieval solution for 10 to 15 min and allow the solution to cool naturally. Incubate the sections in a pre-hybridization solution at 37 ˚C for 1 h. Use Cy5-labeled probe EUB338 5'-GCTGCCTCCCGTAGGAGT-3' (Seebio, ECA0016A, 50 μL) and Cy5-labeled *Prevotella* probe 5'- GCA CCT TCG AGC TTA AGC GT -3' (custom-synthesized) overnight at 37 ˚C. Wash with 2× saline sodium citrate at 37 ˚C for 10 min, 1× SSC at 37 ˚C for 5 min (two changes), and 0.5× SSC at room temperature for 10 min. Counterstain cell nuclei with DAPI (2 μg/μL) for 8 min in the dark. Mount the sections with fade-resistant Mountant. The signal was captured using a PANNORAMIC MIDI digital slide scanner (3DHISTECH Ltd., Budapest, Hungary). Fluorescence images were viewed using CaseViewer version 2.4 (3DHISTECH Ltd., Budapest, Hungary), and the fluorescence intensity was quantified as integrated optical density (IOD) 25, 26 using Image Pro Plus 6.0 software (Media Cybernetics, Silver Spring, MD, USA).

### Weighted gene co-expression network analysis (WGCNA)

WGCNA aims to mine functionally related bacteria with similar co-expression patterns. By calculating the expression correlation coefficients, bacteria with highly correlated expression profiles are clustered into the same module, thereby revealing similar expression patterns. These modules often consist of bacteria that are potentially involved in the same biological processes or pathways. To achieve this, we selected a soft-thresholding power of 8 to ensure a scale-free network, enabling dynamic pruning of dendrogram branches according to cluster morphology. To reduce the likelihood of spurious associations during module identification, the adjacency matrix was subsequently transformed into a Topological Overlap Matrix (TOM). The bacteria within the identified modules were then mapped to construct co-occurrence networks of tissue bacterial communities, which were visualized using Cytoscape 3.5.1 software. Additionally, we explored the correlation between WGCNA modules and various clinical variables, such as age, gender, smoking status, alcohol consumption, histological type, TNM stage, lymph node metastasis, PD-L1 expression, and sample type-by generating heatmaps that illustrate the relationships and corresponding *P*-values for each module.

### RNA extraction and RNA-seq

Total RNA was extracted using the TRIzol reagent (Invitrogen, 15596026CN) according to the manufacturer's instructions. RNA purity and concentration were assessed with a NanoDrop 2000 spectrophotometer (Thermo Scientific, USA), while RNA integrity was evaluated using an Agilent 2100 Bioanalyzer (Agilent Technologies, Santa Clara, CA, USA). Subsequently, transcriptome libraries were constructed following the protocol of the VAHTS Universal V6 RNA-seq Library Prep Kit. RNA sequencing was performed by OE Biotech Co., Ltd. (Shanghai, China) using the Illumina NovaSeq 6000 platform, generating 150 bp paired-end reads. The raw reads in fastq format were processed with fastp to remove low-quality reads, yielding clean reads for subsequent analysis. Alignment to the reference genome (GRCh38) was conducted using HISAT2, followed by quantification of gene expression levels (FPKM). Gene-level read counts were obtained using HTSeq-count.

### Construction of microbe-gene dependency network dependent on conditional mutual information algorithm (CMI)

**Data pre-processing.** For all species-level microbes, only those with abundance in at least 5 samples were retained for subsequent analysis. The same processing was applied to gene. Next, each microbe (gene) was discretized using its median value across all samples as the threshold, setting values below the median to 0 and those equal to or above the median to 1. For phenotype data, when the phenotype is adenocarcinoma vs. squamous cell carcinoma, adenocarcinoma was set to 0 and squamous cell carcinoma to 1. When the phenotype is cancer vs. control, cancer was set to 1 and control to 0. **Calculation of dependency value.** For each microbe and gene pair, conditional mutual information was applied to calculate the dependency value of the gene (denoted as *G*) on the microbe (denoted as *M*) in the context of phenotype (denoted as *P*). The dependency relationship of gene *G* on microbe *M* was calculated via the CMI described as the following equation:



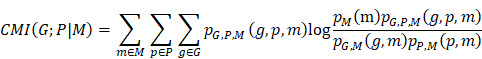



**Significance test of the dependency relation**. We used permutation method to evaluate the significance of every microbe-gene dependency relation. For each candidate pair (*M_i_, G_j_*), we first calculated its real *CMI* value as described above. Then we randomly permuted the abundance of microbe *M_i_* 1000 times and calculated *1,000 CMI* values as the null hypothesis distribution. Then the order (descending) of the real CMI value in the null hypothesis distribution divided by *1000* was taken as the *P*-value of the dependency pair. Finally, we used a threshold (0.05 in this work) to decide whether the pair was significant or not. **Construction of microbe-gene dependency network**. The significant microbe-gene dependency pairs were preserved to construct the dependency network, in which, nodes are microbes and genes, and the edge represents the dependency relationship of the gene on the corresponding microbe.

### Identification of key bacteria by random forest

Random forest is a classic regression and classification ensemble algorithm. By constructing multiple decision trees, each using a different subset of features during training, and by repeatedly evaluating the significance of features in different decision trees, random forest is able to identify robust features that show significance across multiple trees 27. In this study, the randomForest package was used to perform random forest analysis to identify key bacteria (ntree = 100).

### Enrichment analysis of characteristic bacteria

We input the dependent genes of *Bradyrhizobium* and *Prevotella* screened by CMI into the g:Profiler analysis tool (https://biit.cs.ut.ee/gprofiler/gost), selected GO, KEGG, REACTOME and WikiPathway as the background gene set, and used the BH method for *P* value correction. Entries with *P* < 0.05 were considered significantly enriched.

### Immune infiltration correlation analysis

To evaluate the correlation between identified key bacteria and immune cell infiltration, we first calculated cell infiltration scores using the single-sample Gene Set Enrichment Analysis (ssGSEA) algorithm based on our bulk RNA-seq data. A set of 28 immune cell gene markers, derived from the study by Charoentong p *et al.* 28, was employed as the background gene set. Subsequently, Spearman correlation analysis was performed to compute the correlation coefficients and significance between bacterial abundance and immune cell infiltration scores. Finally, the results were visualized using the R package "pheatmap".

### Survival analysis

To assess the association between *Bradyrhizobium* and *Prevotella* and patient survival, we utilized TCGA-LUAD/LUSC data pre-aligned with bacterial sequences, as reported by Chen *et al*. 29. We extracted normal tissue samples (as both bacteria were found to be highly enriched in adjacent non-cancerous tissues) and grouped them based on the median bacterial abundance. Survival analysis was conducted using the R package "survival" and "survminer", with significance evaluated by the log-rank test, where a *P*-value < 0.05 was considered indicative of significant survival differences between the two patient groups.

### Differential gene expression and enrichment analysis

To further validate the association between *Prevotella* and immune regulation within TCGA data, we first grouped the TCGA-LUAD/LUSC adjacent non-cancerous samples according to the median abundance of *Prevotella*. Differential gene expression analysis was then performed using the R package "edgeR". Subsequently, the top 50 genes highly expressed in the high-*Prevotella* group were subjected to enrichment analysis employing the same methods as described earlier.

### Gene set enrichment analysis (GSEA)

The genes identified from the differential expression analysis were ranked by log fold change (logFC) values, from highest to lowest, and used as input for GSEA. The hallmark gene sets (H: hallmark gene sets) were downloaded from the MsigDB database (https://www.gsea-msigdb.org/gsea/msigdb) to serve as the background gene set. GSEA was conducted using the R package "clusterProfiler", with pathways showing a corrected *P*-value < 0.05 and a normalized enrichment score (NES) greater than 0 considered significantly upregulated in the high-*Prevotella* group.

### Generalized linear mixed model and combined diagnostic model construction

To identify biomarkers associated with response to immunotherapy or targeted therapy, a generalized linear mixed model (GLMM) was constructed using the glm function in R. The association between each biomarker and treatment response was evaluated by calculating the odds ratio (OR), where an OR > 1 indicated a positive association with treatment response and an OR < 1 indicated an association with non-response. Biomarkers with a *P* value < 0.05 were considered significant. Receiver operating characteristic (ROC) curves for individual biomarkers were generated using the R package pROC, and AUC was calculated to assess their diagnostic performance in predicting treatment response. To evaluate the diagnostic performance of multiple biomarkers in combination, those identified by GLMM were incorporated into a binary logistic regression model. The immunotherapy cohort was randomly divided into a training set and a validation set in an 8:2 ratio, and ROC analysis was performed to assess the diagnostic performance in both sets.

### Statistical analysis

All statistical analyzes were conducted using R software (version 4.3.0) and Sangerbox (http://sangerbox.com/login.html). For group comparisons of continuous variables following a normal distribution, *t*-tests were used; otherwise, the Wilcoxon test was applied. *P*-values for gene set enrichment analysis were adjusted using the Benjamini-Hochberg method. Survival analysis was performed using the log-rank test, and *P*-values were corrected using the Bonferroni method. Correlations between variables were assessed using Spearman's correlation coefficients. All statistical tests were two-sided, with a threshold of *P* < 0.05 set for statistical significance.

## Results

### Microbial diversity and community structure in patients with NSCLC

**Figure 1:**
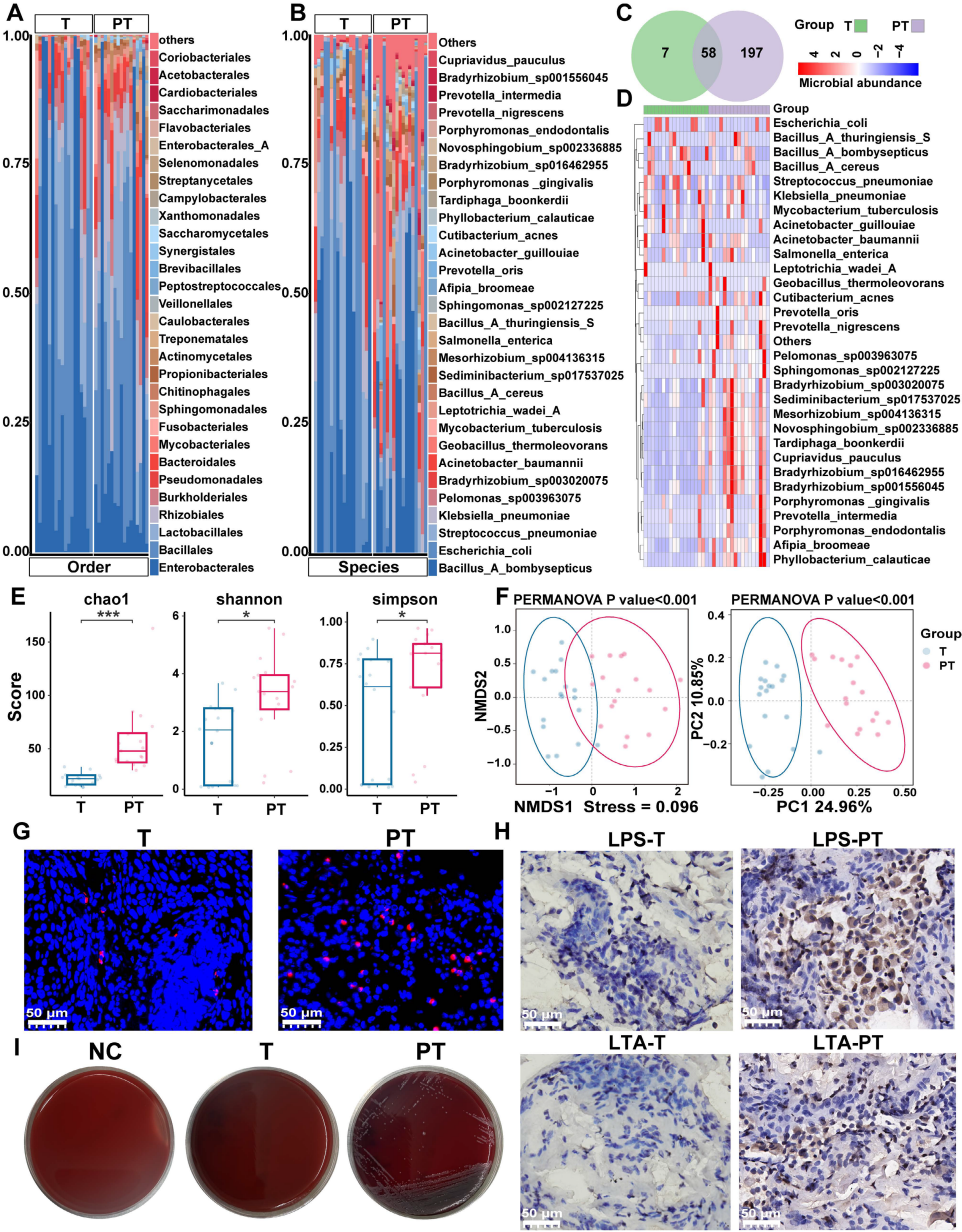
Analysis and validation of the composition of microbiota in NSCLC tissues. (**A**, **B**) Composition features of the microbiota in tumor (T) and paracancerous tissue (PT) groups at the order (**A**) and species (**B**) level. (**C**) A Venn diagram exhibited the shared and unique species between the T and PT groups. (**D**) Abundance of the microbiota in the T and PT groups at the species level. (**E**) Comparison of alpha diversity (Chao1, Shannon index, and Simpson index) between the T and PT groups, Statistical significance was determined by two-sided Wilcoxon rank-sum test, *P < 0.05, ***P < 0.001. (**F**) Comparison of β-diversity between the T and PT groups based on the Binary Jaccard distance. Statistical significance was assessed using PERMANOVA performed with the ADONIS function, *P* < 0.001. (**G**) Results of FISH fluorescence staining NSCLC tissues. The red signal indicates the positive signal of the synthetic FISH probe (EUB338). (**H**) Results of IHC staining of T and PT sections. T: tumor tissue; PT: paracancerous tissue. (**I**) Fresh T and PT of NSCLC patients were used to homogenize and culture live bacteria. NC: negative control.

Twenty paired T and PT samples were collected from patients diagnosed with NSCLC. Following quality control filtering, 17 PT and 18 T samples were included in the final sequencing analysis. Detailed clinical information for all patients is provided in **Table S3**. The 2bRAD-M technology was employed to investigate microbial communities within the clinical samples, yielding 277.24 million raw reads, 247.98 million enzyme reads, and 232.34 million clean reads. On average, each sample yielded 6.64 million clean reads (**Table S4**). Taxonomic classification of the clean reads performed against the 2b-Tag-DB identified 262 species, 111 genera, 62 families, and 37 orders (**Table S5**). The predominant bacterial orders included *Enterobacterales*, *Bacillales*, and *Lactobacillales*, with *Bacillus_A_bombysepticus*, *Escherichia coli*, and *Streptococcus pneumoniae* being the most abundant species (**Figure 1A**-**B**).

At the species level, 58 bacterial taxa were shared between the T and PT groups, with seven species unique to the T group and 197 unique to the PT group (**Figure 1C-D**). Alpha diversity metrics, including the Chao1, Shannon, and Simpson indices, revealed significantly higher bacterial diversity in PT than in T (**Figure 1E**). Beta diversity analysis based on binary Jaccard distance using both NMDS and PCoA revealed a clear separation of microbial communities between T and PT tissue (**Figure 1F**). Furthermore, PERMANOVA performed by the ADONIS test yielded a p-value < 0.001, underscoring the distinct microbial community structures between the two groups (**Figure 1F**).

Based on our sequencing data, we confirmed the presence of bacterial DNA in NSCLC tissues, designed probes targeting pan-bacterial DNA, and performed fluorescence in situ hybridization (FISH). The results showed that the presence of bacteria was higher in NSCLC tissues and in the PT group than in the T group (**Figure 1G**). Immunohistochemical (IHC) staining for LTA and LPS produced similar results (**Figure 1H**).

**Figure 2:**
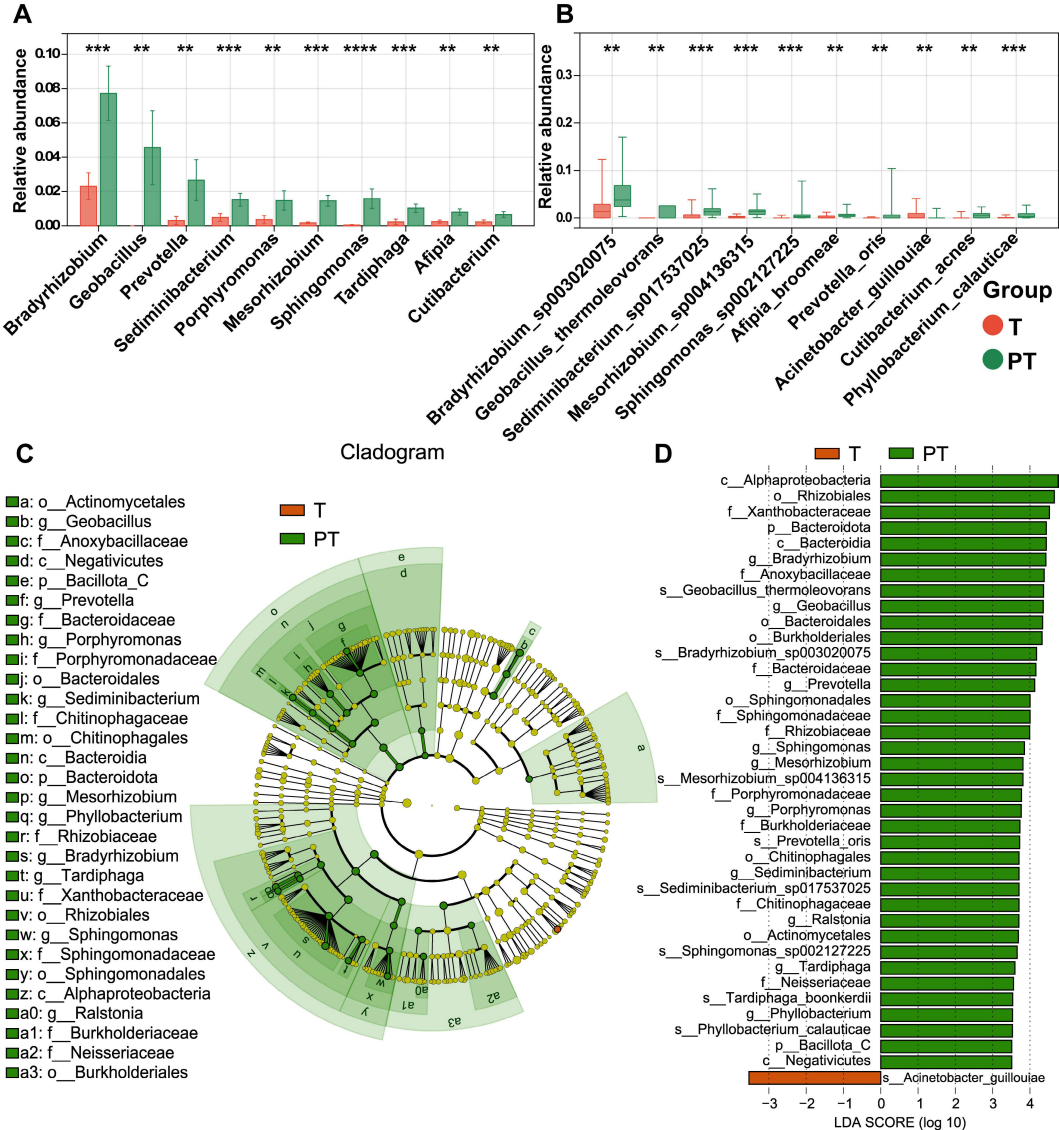
Differential abundances of bacterial taxa between the tumor (T) and paracancerous tissue (PT) groups. (**A**, **B**) The boxplot showed the relative abundance of top ten genus(a) and species (b) were increased in the PT group as determined by two-sided paired Wilcoxon signed-rank tests, *P* < 0.05 considered statistically significant. *P < 0.05, **P < 0.01, ***P < 0.001, ****P < 0.0001. (**C**) Cladogram generated by the LEfSe represents the taxonomic hierarchical structure of the identified microbial populations. Red nodes and green nodes represent relatively high abundance of species with significant difference in T and PT group, respectively. Yellow nodes indicate that there was no significant difference in the comparison of species in the two groups. (**D**) The histogram of LDA score showed 39 biomarkers with significant differences between the T and PT group. LDA score represented the influencing degree of biomarkers.

Next, we performed bacterial culture techniques on five pairs of fresh T and PT samples to verify the presence of viable bacteria. Following homogenization, the samples were evenly spread on culture plates and incubated, resulting in visible colony growth of bacterial colonies in 80% of PT samples, thereby confirming the presence of live bacteria in the lung tissue (**Figure 1I**). Multiple colonies were isolated and identified by MS, confirming the presence of *Bacillus cereus* in fresh PT samples. This finding was consistent with our 2bRAD-M sequencing data. By contrast, no colonies were observed on plates coated with T samples, likely due to the extremely low bacterial abundance in T samples, which was insufficient for visible culture growth. This observation aligned with our sequencing data, which indicated a significantly higher bacterial abundance in the PT group than in the T group (**Figure 2A-B, Table S5**).

Given the marked differences in the tumor microenvironments of LUAD and LUSC tissues, we further examined the compositional diversity of bacterial microbiota. LUAD tissues exhibited greater bacterial species richness than LUSC tissues, whereas no significant differences were observed in the overall community diversity between the two subtypes (**Figure S1A-D**). Moreover, beta diversity analysis revealed no significant differences between the LUAD and LUSC microbiomes (**Figure S1E**).

Overall, these findings indicate that bacterial diversity is significantly higher in PT samples than in T samples of patients with NSCLC, with the presence of viable bacteria further confirmed in PT samples. By contrast, no significant differences in bacterial diversity were observed between LUAD and LUSC tissues.

### Differential abundances in bacterial taxa between T and PT of patients with NSCLC

To identify differentially represented taxa in T and PT samples of patients with NSCLC, the relative abundances of microbial communities were compared between the two groups. The analysis revealed that nearly all of the top 10 differential microbiotas were significantly enriched in the PT group. At the genus level, *Bradyrhizobium*, *Geobacillus*, *Prevotella*, *Sediminibacterium*, and *Porphyromonas* exhibited pronounced enrichment in PT (**Figure 2A**). At the species level, *Bradyrhizobium sp003020075*, *Geobacillus thermoleovorans*, *Sediminibacterium sp017537025*, *Mesorhizobium sp004136315*, and *Sphingomonas sp002127225* were more abundant in PT (**Figure 2B**).

To further identify high-dimensional biomarkers, LEfSe and calculated LDA scores were employed to compare the bacterial taxa between the groups. The resulting cladogram depicting the phylogenetic distribution indicated that biomarkers distinguishing T from PT were predominantly located in the PT group, likely reflecting the significantly higher bacterial abundance in PT samples than in T samples (**Figure 2C**). LDA revealed 39 discriminative features with significantly distinct relative abundances between the groups. Among these, *Bradyrhizobium*, *Geobacillus*, and *Prevotella* emerged as the top three distinguishing genera in PT samples, whereas *Acinetobacter guillouiae* was the most distinctive taxon in T samples (**Figure 2D**). Conversely, only three genera and five species exhibited differential abundance between LUAD and LUSC, with nearly all taxa showing a significantly higher enrichment in the PT group (**Figure S2A-B**). Among these taxa, *Cupriavidus metallidurans* emerged as the most discriminative in LUSC, whereas *Sphingomonadaceae* was the most discriminative in LUAD (**Figure S2C-D**).

These findings indicate that major differences in tissue-resident microbial composition were primarily observed between T and PT, whereas microbial variation between LUAD and LUSC was comparatively limited.

### Correlation between tissue microbial signatures and clinical characteristics

**Figure 3:**
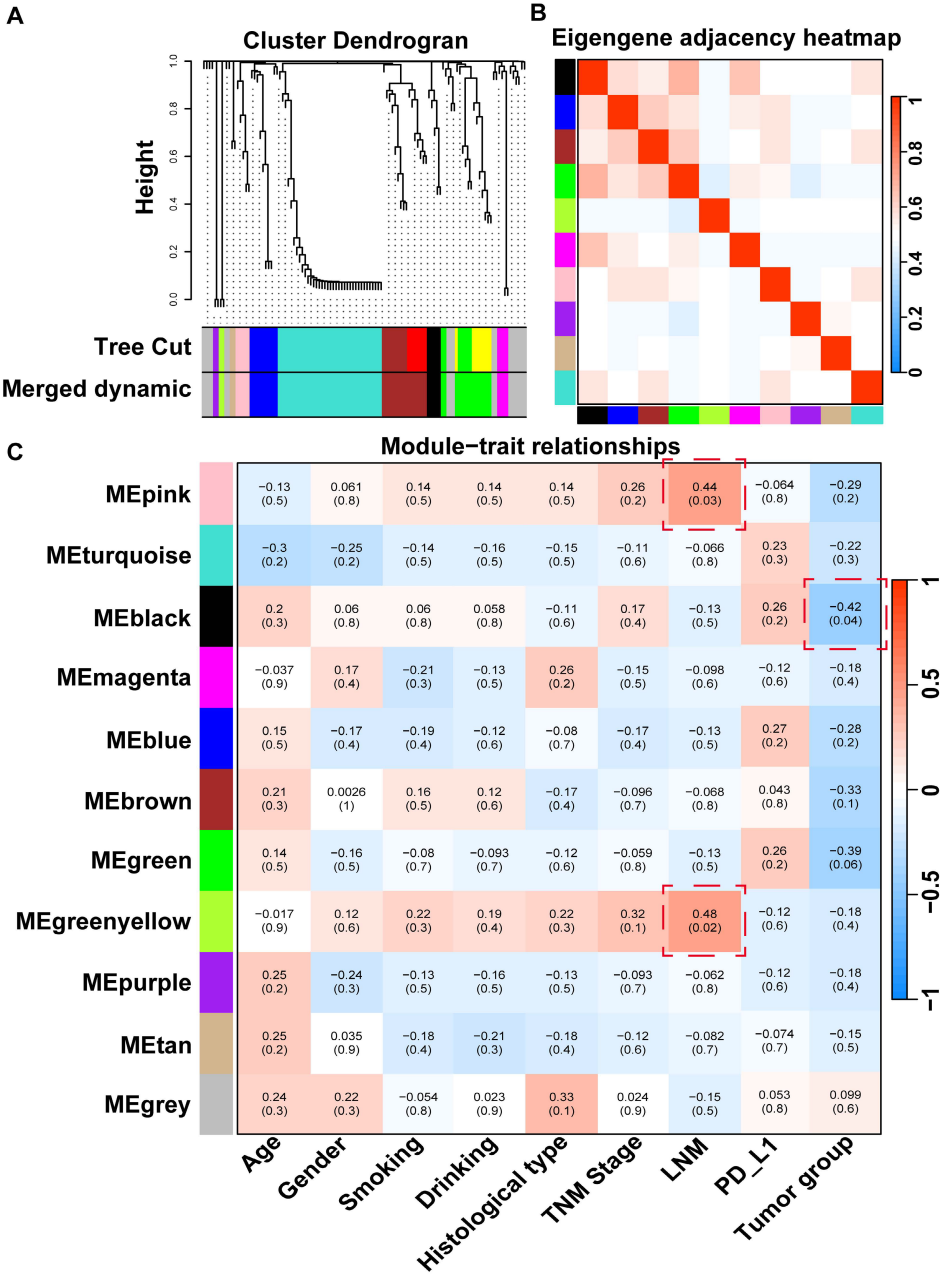
WGCNA analysis of tissue microbes at the genus level. (**A**) Hierarchical clustering dendrogram of co-expressed microbes after module fusion. (**B**) Eigen microbe adjacency correlation heatmap of the function module. (**C**) Heatmap of the correlation between module and clinical trait.

To investigate co-abundant interactions among microbiota and their clinical relevance, WGCNA was employed to characterize the network architecture of tissue-resident microbiota. This method enables the systematic identification of associations between microbial co-abundance modules and clinical traits while preserving sensitivity to low-abundance taxa and minimizing information loss 30. The analysis revealed 11 distinct microbial modules (**Table S6**), with the gray module classified as non-functional and the remaining modules considered functionally relevant (**Figure 3A**). Notable heterogeneity was observed across these functional modules (**Figure 3B**).

Subsequent analyzes evaluated correlations between the identified microbial modules and a range of clinical and pathological characteristics, including age, sex, smoking status, drinking habits, histological type, TNM stage, lymph node metastasis (LNM), PD-L1 expression, and tumor group (**Figure 3C**). The microbial members of the black module were significantly negatively correlated with those of the T group, suggesting a potential role for probiotic taxa with anticancer properties. This effect is likely attributable to the high enrichment of *Geobacillus* within this module, which has been shown to exert antitumor effects via the secretion of the metabolite L-norleucine 31. Additionally, pink and green-yellow modules were significantly positively correlated with tumor LNM, potentially due to the presence of *Veillonella*, a key microbial flora in the lower respiratory tract known to promote tumor LNM in patients with lung cancer 32. In summary, WGCNA provided a comprehensive overview of the complex microbial interactions within NSCLC tissues and revealed strong associations with clinical characteristics.

### Tumor transcriptome and biological status are shaped by tissue-resident microbiota via the tumor immune microenvironment

**Figure 4:**
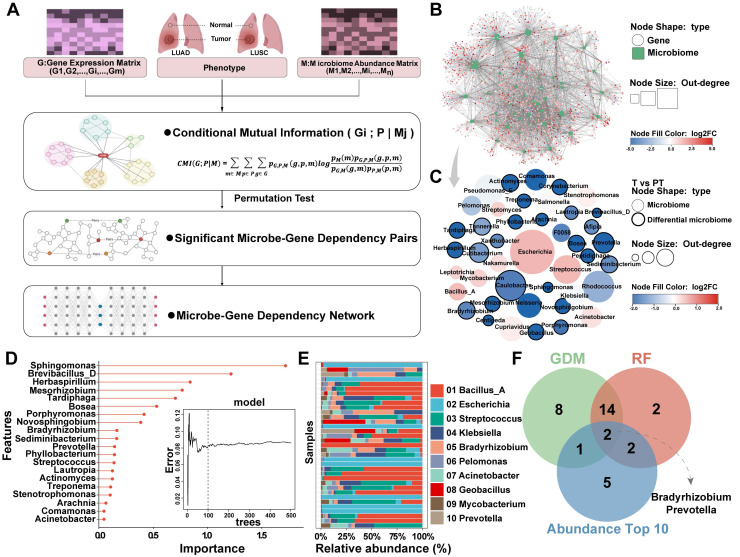
Screening of NSCLC gene-dependent microbes based on CMI technology. (**A**) The construction process of interaction network based on CMI technology. (**B**) Microbes-host interaction network constructed using CMI technology. (**C**) 25 of the 43 gene-dependent genera in the microbes-host interaction network differed between the T and PT groups. (**D**) Random forest was used to screen top 20 microbe variables, 100 trees were selected to build a robust model, and microbes were sorted according to mean decrease accuracy and mean decrease Gini. (**E**) The top 10 abundant genera in tumor (T) and paracancerous tissue (PT) groups. (**F**) Venn diagram showing shared and unique genera between (**C**), (**D**), and (**E**).

The marked enrichment of microbiota in PT prompted us to investigate the association between bacterial abundance and tumor progression, as well as the potential influence of the microbiome on host gene expression and biological processes. To elucidate these interactions, bulk RNA sequencing was performed on tissue samples paired with microbiome data, followed by integrative multi-omics analysis. To more accurately characterize microbe-host interactions, a CMI-based approach was used (**Figure 4A**). The CMI was calculated between phenotypes (T vs. PT) and gene expression profiles, conditioned on the abundance of phenotype-associated microbes, that is, CMI (gene, phenotypes, and microbes). This metric quantifies the additional predictive value of microbial abundance that contributes to phenotypic differentiation through gene expression, reflecting the extent to which gene expression depends on the presence of microbes.

Based on this framework, a microbe-gene interaction network was constructed, leading to the identification of 43 gene-dependent microbes (GDMs) in patients with NSCLC, of which 25 exhibited differential enrichment between T and PT groups (**Figure 4B-C, Table S7**). Random Forest analyzes were then performed to rank the importance of these GDMs, highlighting 20 bacterial taxa with strong discriminative power between T and PT samples (**Figure 4D**). Considering the inherently low abundance of intratumoral microorganisms, the top 10 most enriched bacterial taxa in the tissue samples were selected (**Figure 4E**). These were then intersected with those identified through CMI-based analysis and the top contributors to classification performance, revealing *Bradyrhizobium* and *Prevotella* as representative microbes characterized by high tissue abundance, strong gene dependency, and robust discriminative power (**Figure 4F**).

**Figure 5:**
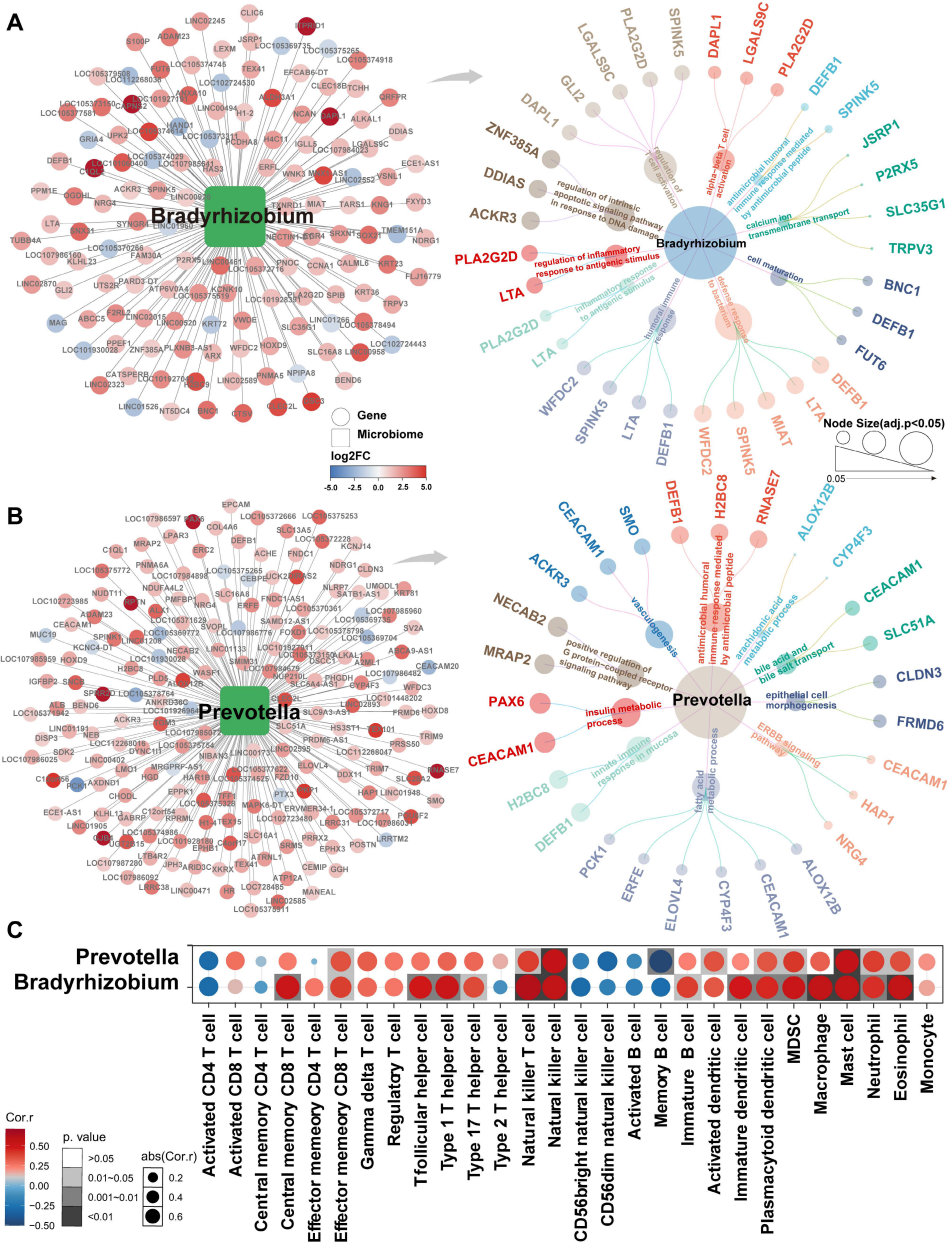
Gene signatures associated with *Bradyrhizobium* and *Prevotella* and their immune infiltration analysis. (**A**) *Bradyrhizobium*-dependent genes and their enrichment pathways (p<0.05). (**B**) *Prevotella*-dependent genes and their enrichment pathways (p<0.05). (**C**) Heatmap of the correlations between *Bradyrhizobium*, *Prevotella* and the infiltration levels of tumor-associated immune cells.

Subsequent analyzes focused on *Bradyrhizobium* and *Prevotella* to investigate their potential functional roles in NSCLC. Pathway enrichment analysis revealed that *Bradyrhizobium*-dependent genes were primarily involved in pathways regulating intrinsic apoptosis signaling in response to DNA damage, inflammatory responses following antigen stimulation, αβ-T-cell activation, and positive regulation of T-cell differentiation within the thymus (**Figure 5A**). By contrast, *Prevotella*-dependent genes were enriched in pathways related to AMP-mediated immune responses, insulin metabolism, and innate immune responses in the mucosa (**Figure 5B**). Notably, both *Bradyrhizobium-* and *Prevotella*-dependent genes were enriched across multiple immune-related pathways. Consequently, we conducted immune cell infiltration analysis, which revealed significant correlations between these bacteria and key components of the tumor immune microenvironment, including effector memory CD8^+^ T cells, natural killer (NK) cells, activated dendritic cells (DCs), macrophages, and neutrophils (**Figure 5C**).

In addition, CMI analysis was extended to various histological subgroups. Within the LUAD and LUSC groups, 24 gene-dependent microorganisms were identified, of which only two (*Novosohingobium and Tardiphaga*) exhibited differential expression between the groups (**Figure S3A-B, Table S8**). Considering the enrichment of microorganisms in PT and the distinct microenvironmental contexts of LUAD and LUSC, separate microbial-gene interaction networks were constructed for LUAD paracancerous (LUADP) and LUSC paracancerous (LUSCP) tissues. A total of 18 gene-dependent microorganisms were identified across these groups. However, none of these microorganisms exhibited significant differences in abundance between LUADP and LUSCP tissues (**Figure S3C-D, Table S9**). Owing to the limited number of differentially gene-dependent microorganisms detected across the LUAD, LUSC, LUADP, and LUSCP groups, no additional subgroup analyzes were conducted. Overall, these findings suggest that tissue microbiota may influence the tumor transcriptome and biological properties by modulating both innate and adaptive immune processes within the tumor microenvironment.

### PT-resident Prevotella is associated with improved prognosis in patients with NSCLC

**Figure 6:**
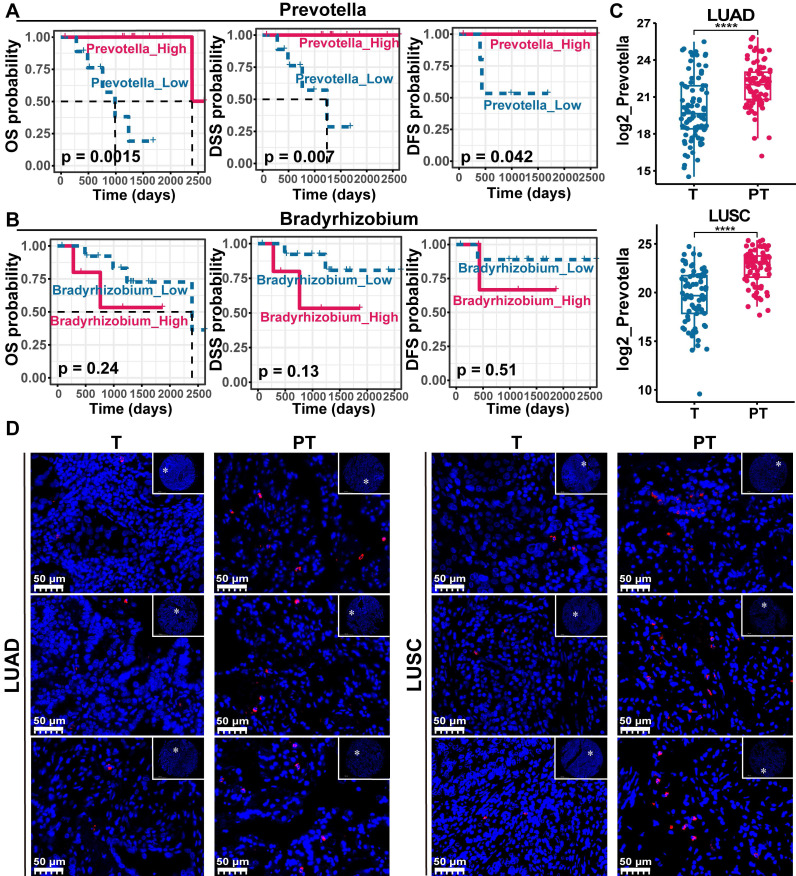
Paracancerous tissue (PT)-resident *Prevotella* is associated with a better prognosis of NSCLC. (**A**, **B**) Kaplan-Meier curves depicting the survival differences of NSCLC patients between high and low paracancerous tissue-resident *Bradyrhizobium* (**A**) and *Prevotella* (**B**) abundance groups based on TCGA data (TCGA-LUAD/LUSC). Survival differences were assessed using the two-sided log-rank test, with *P* < 0.05 considered statistically significant. (**C**) Boxplots show that the abundance of *Prevotella* was higher in PT than in tumor (T) in both LUAD and LUSC as determined by two-sided paired Wilcoxon signed-rank tests, *P* < 0.05 considered statistically significant. (**D**) FISH fluorescence staining images show high enrichment of *Prevotella* in PT than in T in LUAD and LUSC.

Given the significant associations previously identified between *Bradyrhizobium*, *Prevotella*, and antitumor immune components in NSCLC, we hypothesized that these microbial taxa may be linked to improved patient prognosis. Survival analyzes were first performed using PT data from NSCLC cases in the TCGA cohort, in which both *Bradyrhizobium* and *Prevotella* were detected. The results revealed that *Prevotella* abundance was significantly associated with favorable clinical outcomes, including prolonged overall survival (OS), disease-specific survival, and disease-free survival (**Figure 6A**), whereas no significant associations were observed between *Bradyrhizobium* and patient survival outcomes (**Figure 6B**). And our previous WGCNA analysis of the clinical relevance of microbial modules revealed that the turquoise module, which includes *Prevotella*** (Table S6)**, was negatively correlated with TNM stage, LNM, and T group, but positively correlated with PD-L1 expression **(Figure 3)**. This finding further supports the observation that patients with higher *Prevotella* abundance tend to have better prognosis.

**Figure 7:**
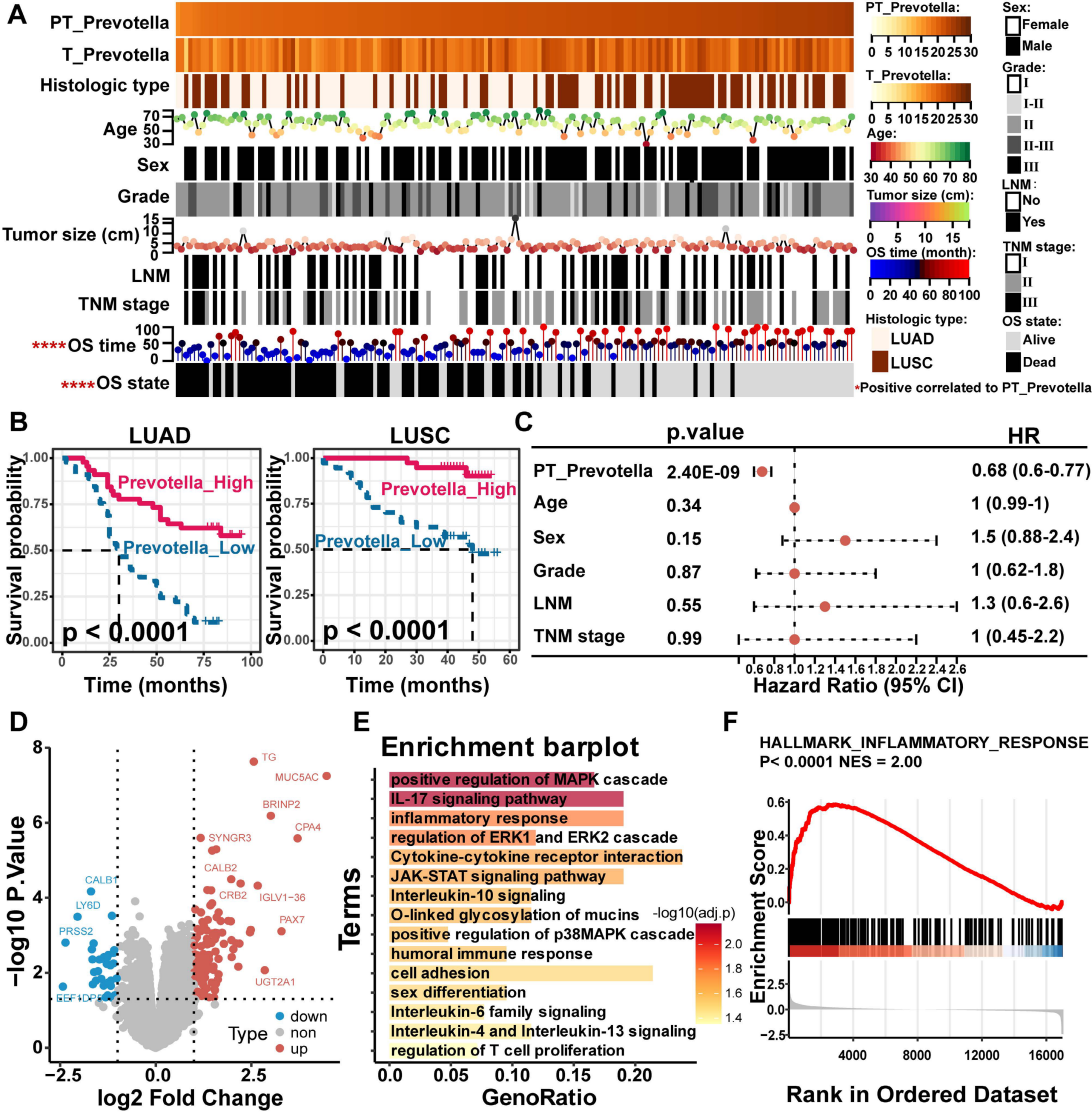
Effect of paracancerous tissue (PT)-resident *Prevotella* on clinical prognosis of NSCLC. (**A**) Heatmap showing the distribution of clinical parameters across samples and their association with the PT-resident *Prevotella*. (**B**) Kaplan-Meier curves show a longer OS in the high PT-resident *Prevotella* group than in the low *Prevotella* group in both LUAD and LUSC. Survival differences were assessed using the two-sided log-rank test, with *P* < 0.05 considered statistically significant. (**C**) Forest plot illustrating hazard ratios (HR) of PT-resident *Prevotella* and other clinical parameters based on multivariable Cox regression analysis. (**D**) Volcano plot showing differential gene expression between the *Prevotella* high- and low-abundance groups, as determined by edgeR. Genes with *P* < 0.05 were considered significantly differentially expressed. (**E**) Functional enrichment analysis of highly expressed genes in high abundance group of *Prevotella*. (**F**) GSEA analysis showed that the inflammatory response pathway was significantly enriched in the high abundance group of *Prevotella*.

To validate these findings, *Prevotella* abundance was quantitatively assessed in clinical tissue microarrays comprising 90 LUAD and 75 LUSC samples using FISH. These results were consistent with the sequencing data, which showed significantly higher *Prevotella* abundance in PT samples than in T samples (**Figure 6C-D**). Further clinical correlation analysis revealed a significant positive relationship between *Prevotella* abundance in PT and prolonged OS (**Figure 7A**). Survival analyzes further revealed that patients with high *Prevotella* abundance exhibited markedly improved OS in both LUAD and LUSC (**Figure 7B**). Importantly, multivariate Cox regression analysis adjusted for clinical variables, including age, sex, tumor grade, LNM, and TNM stage, confirmed that *Prevotella* abundance in PT was an independent prognostic factor for patients with NSCLC (**Figure 7C**). Collectively, these results corroborated our initial hypothesis and highlighted *Prevotella* as a promising prognostic biomarker for improving NSCLC outcomes.

To gain deeper insights into the potential mechanisms by which *Prevotella* influences the prognosis of patients with NSCLC, differential gene expression and functional enrichment analyzes were conducted between patients with high and low *Prevotella* abundance in PT. Notably, genes upregulated in patients with high *Prevotella* abundance were significantly enriched in signaling pathways closely associated with immunotherapy and targeted therapy, including the mitogen-activated protein kinase (MAPK) pathway (ERK and p38), JAK-STAT signaling, T-cell proliferation, and inflammatory responses (**Figure 7D-F**). These pathways are well-established regulators of therapeutic efficacy. Collectively, these findings demonstrate that *Prevotella* enrichment in the PT of patients with NSCLC is associated with improved prognosis and serves as an independent prognostic factor. This beneficial effect may be mediated through its modulation of host responses to targeted therapy and immunotherapy.

### PT-resident Prevotella combined with routine blood indicators may serve as a predictive biomarker for immunotherapy response in NSCLC

**Figure 8:**
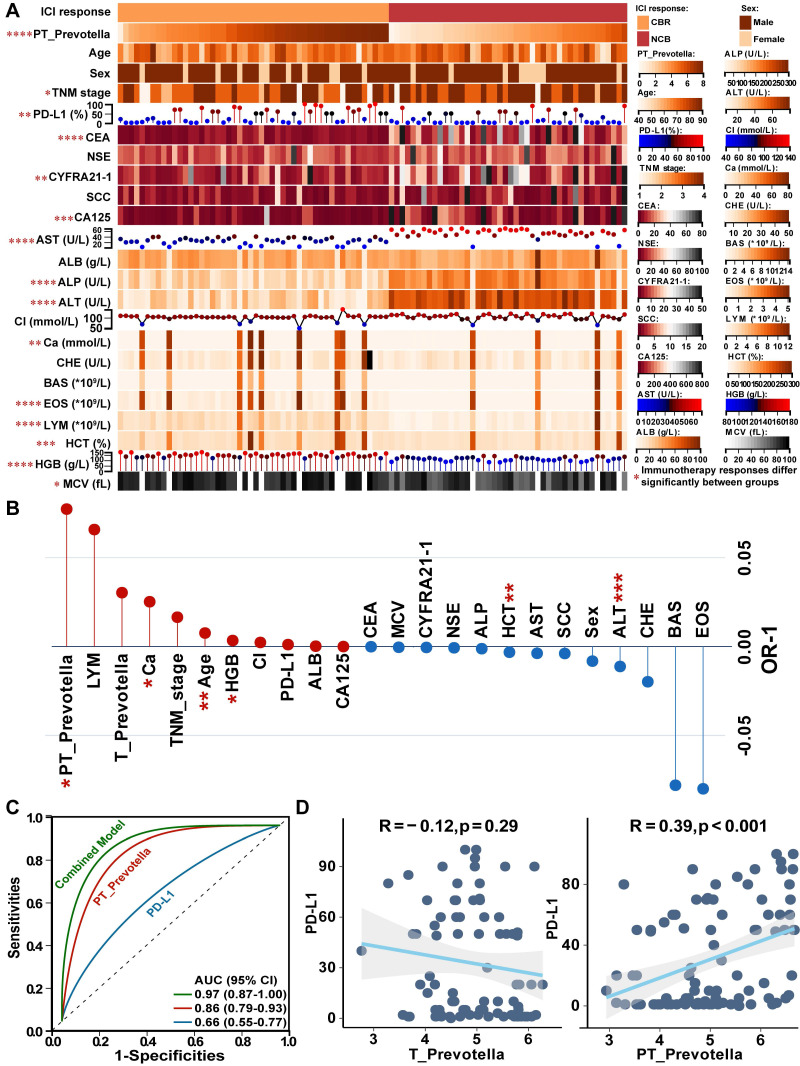
Effect of paracancerous tissue (PT)-resident* Prevotella* on immunotherapy response of NSCLC. (**A**) The heatmap displays the distribution of PT-resident *Prevotella* and clinical indicators between the CBR and NCB groups in the immunotherapy cohort. (**B**) The Generalized Linear Mixed Model (GLMM) identifies independent indicators associated with immunotherapy response. (**C**) The ROC curve for predicting immunotherapy response using the combined diagnostic model, PT-resident *Prevotella*, and PD-L1. (**D**) Correlation between tumor (T)- and PT-resident *Prevotella* and PD-L1 expression, assessed using two-sided Spearman's rank correlation. *P* < 0.05 was considered statistically significant.

To investigate the association between *Prevotella* and immunotherapy response, 94 patients treated with anti-PD-L1 therapy were enrolled from two independent centers. According to RECIST v1.1 criteria, 50 patients exhibited a clinical benefit response (CBR), whereas 44 showed no clinical benefit (NCB) (**Figure 8A**). Notably, significant differences were observed between the CBR and NCB groups in terms of PT-resident *Prevotella*, TNM stage, PD-L1 expression, commonly used lung cancer biomarkers (CEA, CYFRA21-1, and CA125), and routine blood tests indicators (including aspartate aminotransferase [AST], alkaline phosphatase [ALP], alanine aminotransferase [ALT], calcium [Ca], eosinophil [EOS], lymphocytes [LYM], hematocrit [HCT], hemoglobin [HGB], and mean corpuscular volume [MCV]) **(Figure 8A)**. To assess the independent predictive value of these clinical variables, a generalized linear mixed model was constructed incorporating all clinical indicators. After adjustment, PT-resident *Prevotella*, Ca, age, and HGB were identified as independent protective factors, whereas HCT and ALT were identified as independent risk factors for immunotherapy response (**Figure 8B, Table S10**). Based on these predictors, a combined predictive model was developed by integrating PT-resident *Prevotella* abundance with routine blood test indicators. The model formula is expressed as follows:

Predictive Score = 9.675 × PT_*Prevotella* + 1.749 × Ca + 8.086 × Age + 3.332 × HGB - 1.676 × HCT - 5.930 × ALT

Patients with a predictive score above the optimal cutoff of 720.7 were classified as likely responders to immunotherapy. The combined diagnostic model demonstrated superior predictive performance (AUC: 0.97) compared with models based solely on PD-L1 expression (AUC: 0.66) or PT-resident *Prevotella* alone (AUC: 0.86) (**Figure 8C**).

Although PD-L1 is widely used as a clinical biomarker, it was not significant as an independent predictor in the multivariate model (**Figure 8B**), suggesting that its predictive utility may be modulated by other covariates within the tumor microenvironment. Given the previously observed association between paracancerous *Prevotella* abundance and tumor-infiltrating immune components, further correlation analysis revealed a significant positive association between *Prevotella* abundance and PD-L1 expression (**Figure 8D**). Consistently, IHC staining of NSCLC tissues confirmed that higher *Prevotella* abundance in PT samples corresponded to elevated PD-L1 expression in T samples (**Figure S4**).

To comprehensively evaluate the predictive value of *Prevotella*, an additional cohort of 52 patients with NSCLC receiving *EGFR*-targeted therapy was analyzed (36 vs. 16 in the CBR and NCB groups, respectively). The clinical feature distributions between the groups are shown in **Figure S5A**. A significantly higher abundance of PT-resident *Prevotella* was observed in the CBR group. Although PT-resident *Prevotella* remained an independent predictor of response to targeted therapy after multivariate adjustment (**Figure S5B**) and its AUC exceeded 0.75 (**Figure S5C**), its positive predictive value was only 0.52. Moreover, no significant differences were observed in the *EGFR* mutation status (**Figure S5D**), suggesting that the predictive capacity of PT-resident *Prevotella* for targeted therapy is limited.

In summary, integrating PT-resident *Prevotella* abundance with routine blood test indicators provides a more accurate prediction of immunotherapy response in patients with NSCLC than PD-L1 alone; however, this predictive advantage is not evident in the context of targeted therapy. Moreover, the clinical role of PD-L1 may be influenced by the adjacent paracancerous microbial microenvironment, particularly *Prevotella*.

## Discussion

The introduction of ICIs has significantly improved the survival outcomes for patients with NSCLC and reshaped their treatment landscape 5. Nevertheless, significant interindividual heterogeneity in treatment response and the limited proportion of long-term responders underscore the need for novel biomarkers to optimize patient stratification 6. Tissue-resident microbiota actively participates in tumor immune modulation and holds potential as a powerful indicator of therapeutic efficacy 18, 19. The present study integrated microbiome and transcriptome profiles from paired clinical specimens to construct a microbe-host-gene interaction network, through which *Bradyrhizobium* and *Prevotella* were identified as key taxa exhibiting high host-gene dependency. Notably, *Prevotella* demonstrated strong positive associations with enhanced antitumor immunity and favorable clinical outcomes. Furthermore, a predictive model incorporating PT-resident *Prevotella* abundance with routine blood test indicators exhibited robust predictive performance, offering a novel and practical companion diagnostic tool to facilitate personalized immunotherapy in NSCLC.

While intratumoral microbes are significantly more abundant than those in adjacent non-tumor tissues in liver 33, pancreatic 34, and breast cancers 12, lung cancer exhibits an opposite pattern characterized by substantial microbial accumulation non-tumorous regions, as reflected by markedly reduced microbial abundance and diversity within tumor tissues 19, 35-37. In the present study, 2bRAD-M was employed to profile microbial communities in NSCLC T and PT samples due to its advantage in low-biomass microbiome detection. Several intratumoral bacteria previously reported in NSCLC, including *Acinetobacter*, *Streptococcus*, *Sphingomonas*, and *Pseudomonas* 20, 38, were also detected in our study. Distinct from these prior observations, our data revealed a unique microbial signature in PT samples, marked by higher abundance of *Bradyrhizobium*, *Geobacillus*, *Prevotella*, and other genera, alongside increased microbial diversity compared with T samples. This differential microbial pattern between T and PT samples was further validated by both IHC and FISH analyzes. Moreover, bronchoalveolar lavage fluid, a biofluid in direct contact with the lung cancer microenvironment, exhibits a microbial composition similar to that of tumor tissues 39. The microbial diversity is also markedly reduced compared with that of adjacent non-cancerous and healthy tissues 40, further supporting the presence of characteristic microbial depletion within lung cancer lesions. One possible explanation for the reduced intratumoral microbiota in lung cancer may be attributed to the elevated expression of MUC5AC within adjacent non-cancerous tissues, which, as a mucin, may provide favorable adhesion sites for microbial colonization 36. Collectively, these findings suggest that the transition from normal lung tissue to NSCLC involves substantial remodeling of the microbiome.

To elucidate how intratumoral microbiome remodeling influences NSCLC progression, transcriptomic data were integrated to construct a microbe-gene interaction network, through which *Bradyrhizobium* and *Prevotella* were identified as key bacterial taxa exhibiting the highest gene dependency in NSCLC. These taxa were markedly enriched in PT samples, and their associated genes were significantly involved in immune-related pathways, showing strong correlations with major immune components, including T cells, NK cells, B cells, and DCs. Notably, the pathways identified by the CMI-based analysis of *Prevotella*-dependent genes differed from those enriched in the high-*Prevotella* group in bulk transcriptomic analysis. This difference likely reflects the distinct analytical focuses, as the CMI method captures *Prevotella*-specific regulatory interactions, whereas bulk analysis reveals overall transcriptional changes. These complementary results collectively enhance understanding of how *Prevotella* modulates host immune regulation in NSCLC.

In lung cancer, *Bradyrhizobium* preferentially localizes to non-tumor regions and exhibits higher abundance in early-stage patients compared with late-stage patients 41, 42. This genus has been implicated in suppressing prostate tumor progression by recruiting immune cells and downregulating androgen receptor expression 43. Similarly, *Prevotella* colonizes healthy lung tissue, with a progressive decline in abundance observed during lung cancer development 44, 45. *Prevotella* promotes the regression of high-grade intraepithelial neoplasia via interactions with DCs 46. The observed associations of *Bradyrhizobium* and *Prevotella* with antitumor immunity prompted further evaluation of their prognostic value in NSCLC. Higher *Prevotella* abundance, but not *Bradyrhizobium* abundance, correlated with improved outcomes, a finding that was validated in an independent cohort of 165 cases. Further investigation of a cohort of 94 patients who received immunotherapy demonstrated that the observed prognostic enhancement stemmed from the correlation of *Prevotella* with and favorable therapeutic outcomes. Compared with the conventional biomarker PD-L1, the combination of PT-resident *Prevotella* and routine blood test indicators enhanced the predictive power for NSCLC immunotherapy response, achieving an AUC exceeding 0.95. Previous studies have demonstrated that *Prevotella* is associated with favorable immunotherapy responses in hepatocellular carcinoma 47, gastrointestinal malignancies 48, and hematologic malignancies 49. Therefore, the integrative predictive model developed herein may possess broad applicability and potential for extension to additional cancer types.

Furthermore, the relationship between *Prevotella* and targeted therapy was explored, but its predictive capacity for treatment response proved limited, with a positive predictive value of merely 0.52. Although *Prevotella* was associated with MAPK and JAK-STAT signaling pathways related to *EGFR*-targeted therapy, no significant differences were observed between *EGFR*-mutant and wild-type tumors, highlighting the need for further validation. Notably, although previous studies have implicated the MAPK pathway acts as a potential oncogenic signaling cascade promoting NSCLC progression 50, our study revealed a significant positive correlation between *Prevotella*, identified as a protective factor associated with favorable NSCLC prognosis, and MAPK signaling. This seemingly paradoxical association may be attributed to the fact that MAPK activation is often accompanied by increased infiltration of CD8⁺PD1⁺ T cells and proinflammatory polarization of tumor-associated macrophages, both of which enhance antitumor immune responses 51, 52. In this study, the significant positive correlation observed between *Prevotella* and CD8⁺ T cells may account for its association with MAPK activation, suggesting that the interaction between *Prevotella* and MAPK likely occurs indirectly through CD8⁺ T cells rather than via a direct causal mechanism. This represents an important avenue for future mechanistic investigations. Taken together, despite the need for large-scale clinical validation, *Prevotella* emerges as a promising biomarker candidate for predicting NSCLC response to ICIs rather than to targeted therapies.

Tumor-associated microbes play a pivotal role in modulating host immune responses and influencing the efficacy of cancer immunotherapy. In melanoma, the co-presentation of intratumoral bacteria-derived human leukocyte antigen peptides by both antigen-presenting cells and tumor cells enhances the presentation of immunogenic antigens, thereby promoting T-cell activation and improving the efficacy of ICIs 53. Similarly, intratumoral *Lactobacillus reuteri* and *Fusobacterium nucleatum* have been shown to enhance ICI efficacy by promoting the accumulation of Interferon-gamma-positive CD8⁺ T cells within the tumor microenvironment 54, 55. In addition to NSCLC, *Prevotella* enrichment has been consistently associated with favorable immunotherapy responses across gastrointestinal and hematologic malignancies, correlating with delayed tumor progression, reduced mortality, and higher rates of complete remission 48, 49, 56. These converging observations across cancer types suggest that *Prevotella* plays an active and conserved role in augmenting antitumor immunity, underscoring its potential as a predictive biomarker of ICI efficacy. More importantly, a notable concordance between *Prevotella* abundance and PD-L1 expression patterns was observed in the present study, suggesting that *Prevotella* may act as a potential regulatory factor of PD-L1, which could account for its critical influence on immunotherapy response. A plausible underlying mechanism may involve the ability of *Prevotella*-stimulated bone marrow-derived DCs to prime naïve T-helper cells, leading to up to a fivefold increase in interleukin (IL)-17 levels compared with other commensal bacteria 57. Elevated IL-17 production subsequently upregulates PD-L1 expression in tumor cells, thereby enhancing the efficacy of immunotherapy 58. Further studies are warranted to elucidate the precise molecular mechanisms through which *Prevotella* regulates PD-L1 expression.

The primary strength of this study lies in the use of paired clinical tissue samples, which enabled a comprehensive, system-level analysis of host-intratumoral microbiota interactions through the simultaneous acquisition of both microbiome and host transcriptome datasets. Clinical validation across prognosis, immunotherapy, and targeted therapy cohorts further enhanced the robustness and reliability of the results. Nonetheless, several limitations should be acknowledged. First, *Prevotella* has not yet been specifically cultured from clinical tissues, limiting direct mechanistic validation; second, the clinical relevance of *Bradyrhizobium* was assessed only using public datasets, without confirmation in independent clinical cohorts. Finally, the predictive value of *Prevotella* for immunotherapy efficacy was derived from a relatively small clinical cohort without multicenter validation and its predictive accuracy may vary across different immunotherapeutic agents. Therefore, further large-scale, prospective, multicenter studies are warranted.

## Conclusion

In summary, a comprehensive analysis revealed significant differences in the microbial communities between NSCLC tumors and PT, with PT exhibiting markedly higher microbial richness and diversity. Viable bacteria were successfully isolated and cultured from fresh tissue samples, confirming the presence of live bacteria within lung tissues. Moreover, microbial-host interactions were delineated using the CMI approach, which identified *Bradyrhizobium* and *Prevotella* as key bacteria exhibiting the strongest host-gene dependency in NSCLC. Both taxa were significantly enriched in pathways associated with innate and adaptive immune responses. Notably, *Prevotella* is closely associated with prolonged survival in patients with NSCLC, with its prognostic advantage largely attributable to its influence on immunotherapy responsiveness. In the immunotherapy cohort, a predictive model integrating PT-resident *Prevotella* abundance with routine blood test indicators demonstrated robust performance in forecasting immunotherapy outcomes, highlighting a promising companion diagnostic tool to advance personalized immunotherapy for patients with NSCLC.

## Supplementary Material

Supplementary figures.

Supplementary tables.

## Abbreviations

ICIs: immune checkpoint inhibitors
NSCLC: non-small cell lung cancer
PT: paracancerous tissue
T: tumor tissue
GDM: gene-dependent microbes
IL: interleukin
PD-L1: programmed death-ligand 1
LUAD: lung adenocarcinoma
LUSC: lung squamous cell carcinoma
FISH: fluorescence in situ hybridization
IHC: immunohistochemistry
LNM: lymph node metastasis
OS: overall survival
DSS: disease-specific survival
CBR: clinical benefit response
NCB: no clinical benefit
EGFR: epidermal growth factor receptor
MAPK: mitogen-activated protein kinase
DCs: dendritic cells
NK cells: natural killer cells
AST: aspartate aminotransferase
ALP: alkaline phosphatase
ALT: alanine aminotransferase
Ca: calcium
EOS: eosinophil
LYM: lymphocytes
HCT: hematocrit
HGB: hemoglobin
MCV: mean corpuscular volume
