# Supplementary material for: Tumor microbiome-transcriptome crosstalk identifies Prevotella as an immunotherapeutic predictor in NSCLC
Source: Theranostics. 2026 Jan 1;16(7):3426–46. doi: 10.7150/thno.126091 (PMC12846730; doi:10.7150/thno.126091)
Supplement: Supplementary file 1 — Supplementary figures. [file thnov16p3426s1.pdf]

Supplementary figures

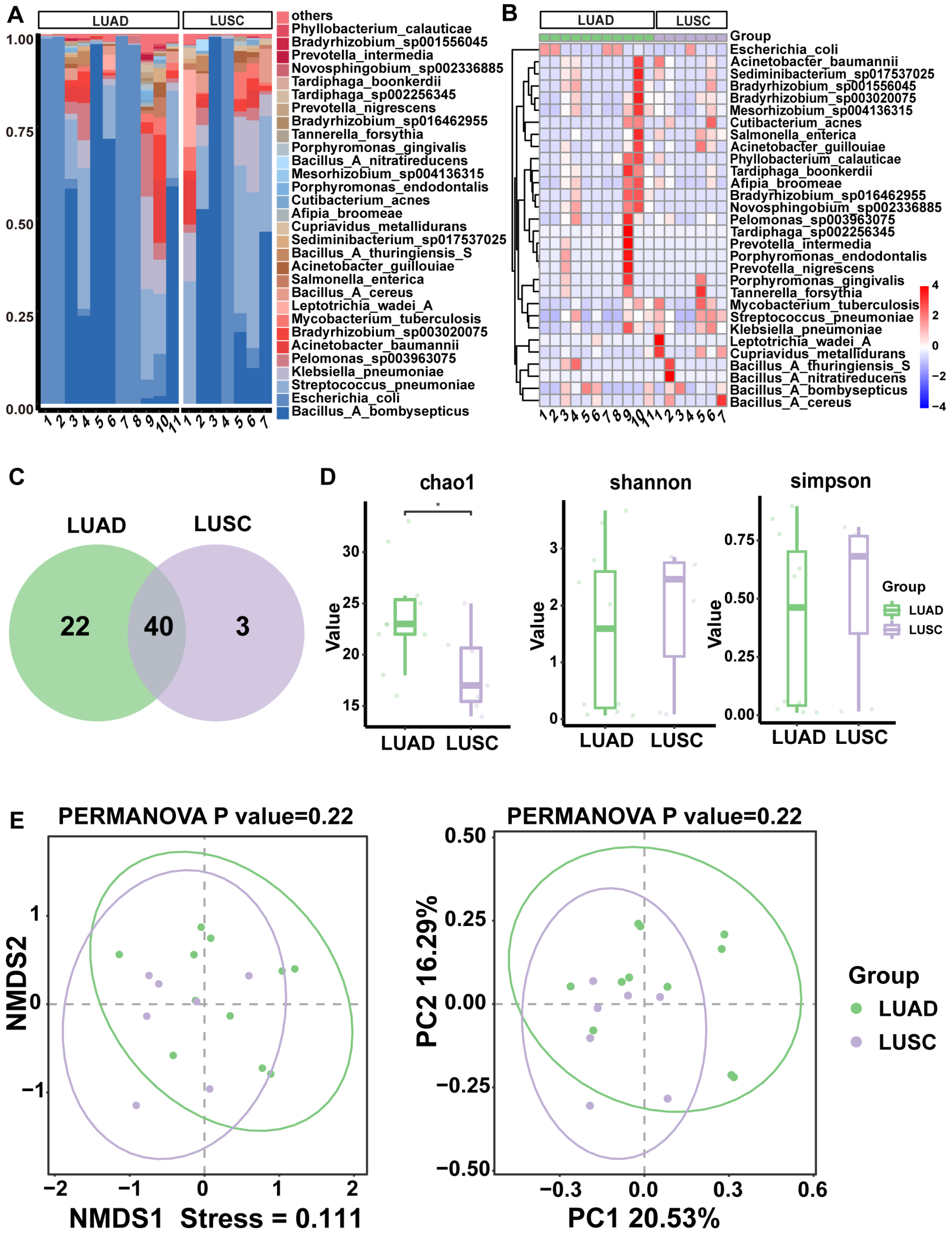

Figure S1

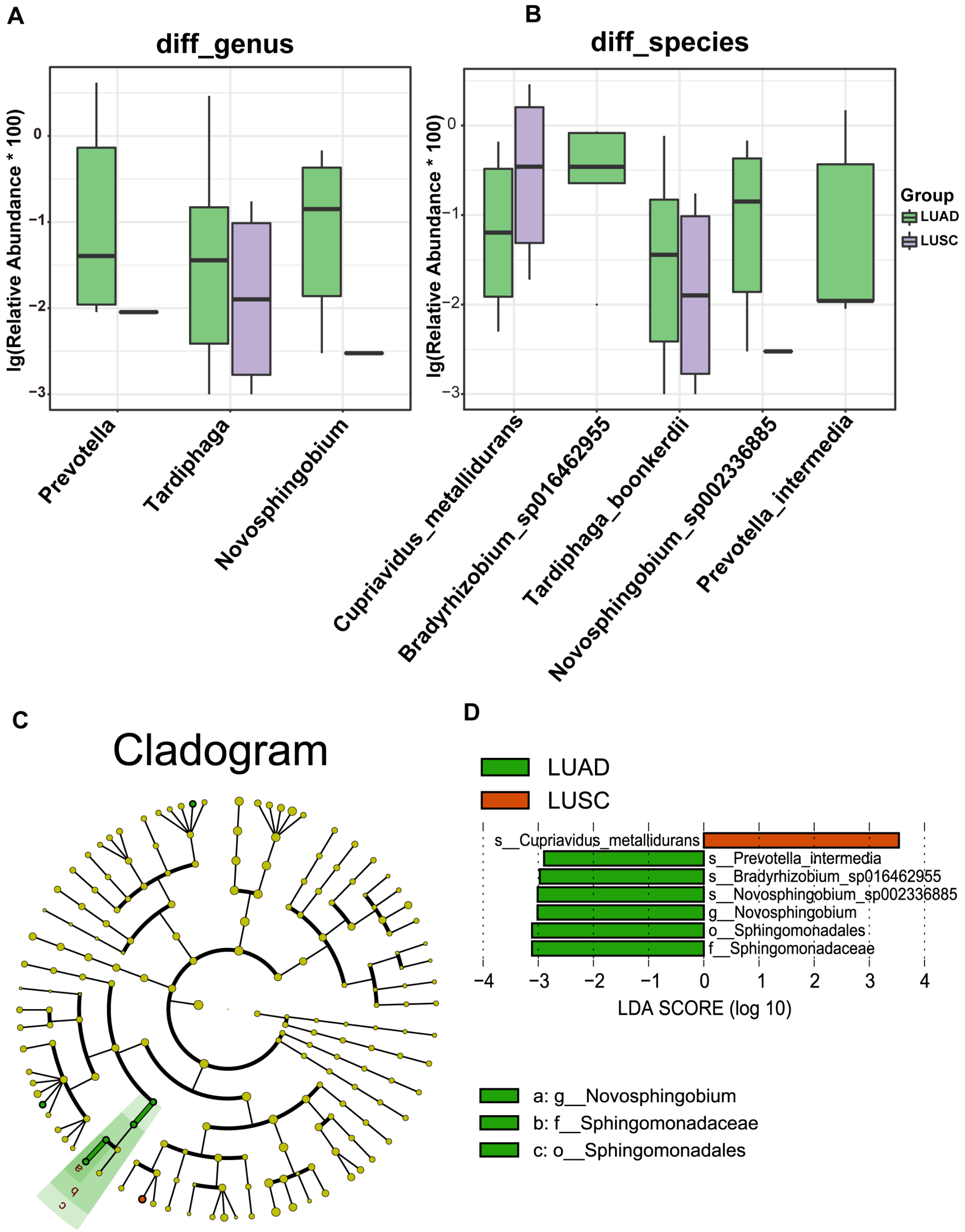
A
diff_genus
lg(Relative Abundance * 100)
0
−1
−2
−3
Prevotella
Tardiphaga
Novosphingobium
B
diff_species
lg(Relative Abundance * 100)
0
−1
−2
−3
Cupriavidus_metallidurans
Bradyrhizobium_sp016462955
Tardiphaga_boonkerdii
Novosphingobium_sp002336885
Prevotella_intermedia
Group
LUAD
LUSC
C
Cladogram
a
b
c
D
LUAD
LUSC
s__Cupriavidus_metallidurans
s__Prevotella_intermedia
s__Bradyrhizobium_sp016462955
s__Novosphingobium_sp002336885
g__Novosphingobium
o__Sphingomonadales
f__Sphingomonadaceae
−4
−3
−2
−1
0
1
2
3
4
LDA SCORE (log 10)
a: g__Novosphingobium
b: f__Sphingomonadaceae
c: o__Sphingomonadales

Figure S2

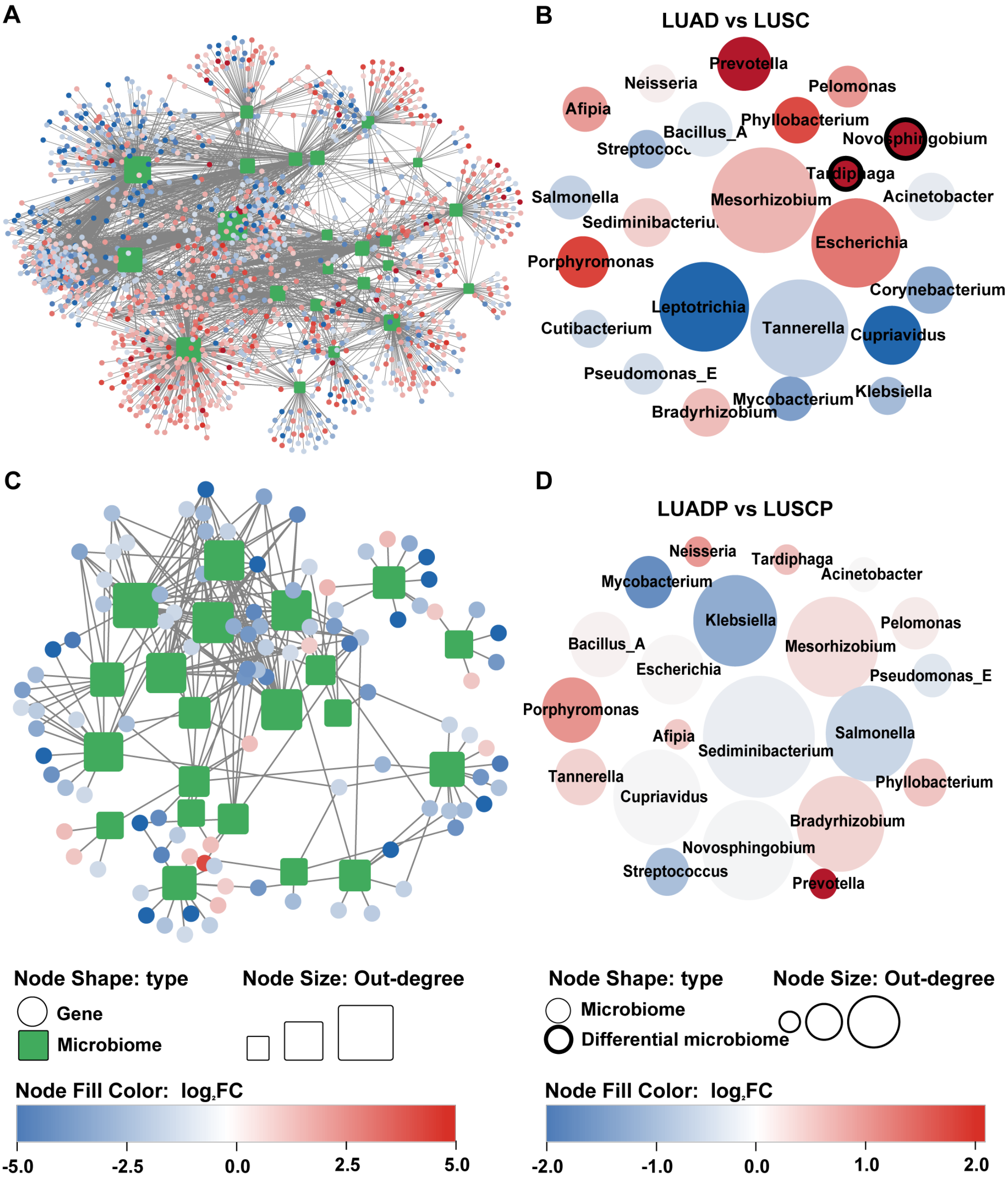
A
B
LUAD vs LUSC
Prevotella
Neisseria
Pelomonas
Afipia
Phyllobacterium
Bacillus_A
Novosphingobium
Tardiphaga
Acinetobacter
Salmonella
Mesorhizobium
Escherichia
Porphyromonas
Corynebacterium
Leptotrichia
Tannerella
Cupriavidus
Cutibacterium
Pseudomonas_E
Mycobacterium
Klebsiella
Bradyrhizobium
C
D
LUADP vs LUSCP
Neisseria
Tardiphaga
Acinetobacter
Mycobacterium
Klebsiella
Pelomonas
Bacillus_A
Mesorhizobium
Escherichia
Pseudomonas_E
Porphyromonas
Afipia
Salmonella
Sediminibacterium
Tannerella
Phyllobacterium
Cupriavidus
Bradyrhizobium
Novosphingobium
Streptococcus
Prevotella
Node Shape: type
Gene
Microbiome
Node Size: Out-degree
Node Fill Color: log2FC
-5.0
-2.5
0.0
2.5
5.0
Node Shape: type
Microbiome
Differential microbiome
Node Size: Out-degree
Node Fill Color: log2FC
-2.0
-1.0
0.0
1.0
2.0

Figure S3

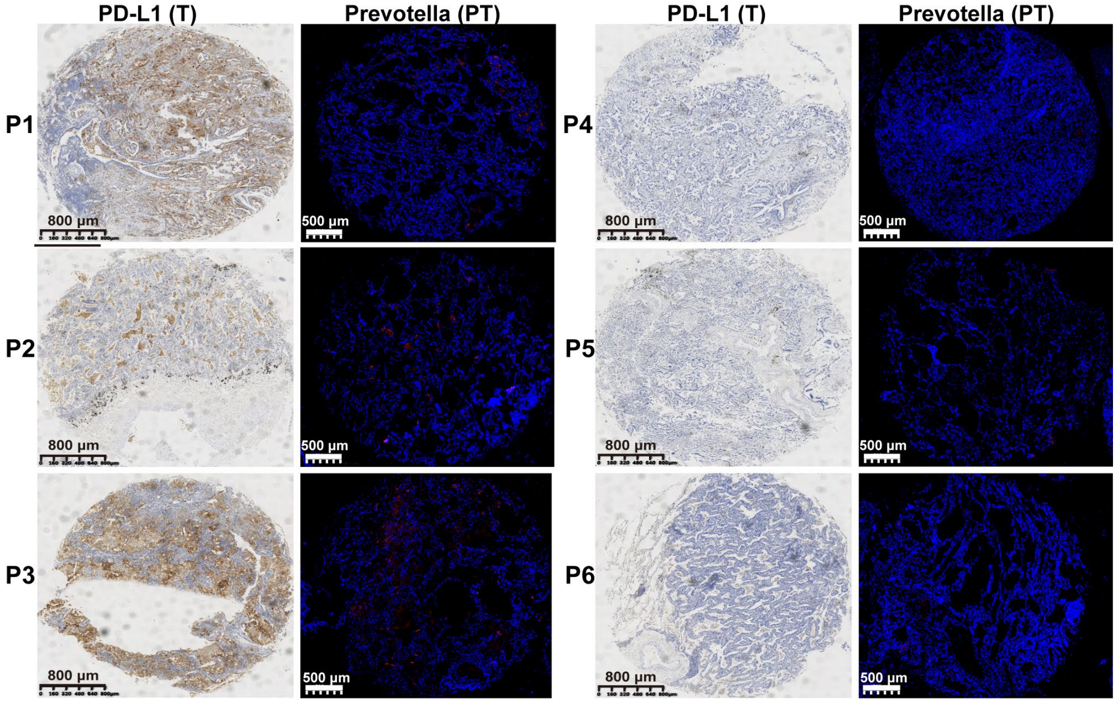
PD-L1 (T)
Prevotella (PT)
PD-L1 (T)
Prevotella (PT)
P1
P2
P3
P4
P5
P6
800 μm
500 μm

Figure S4

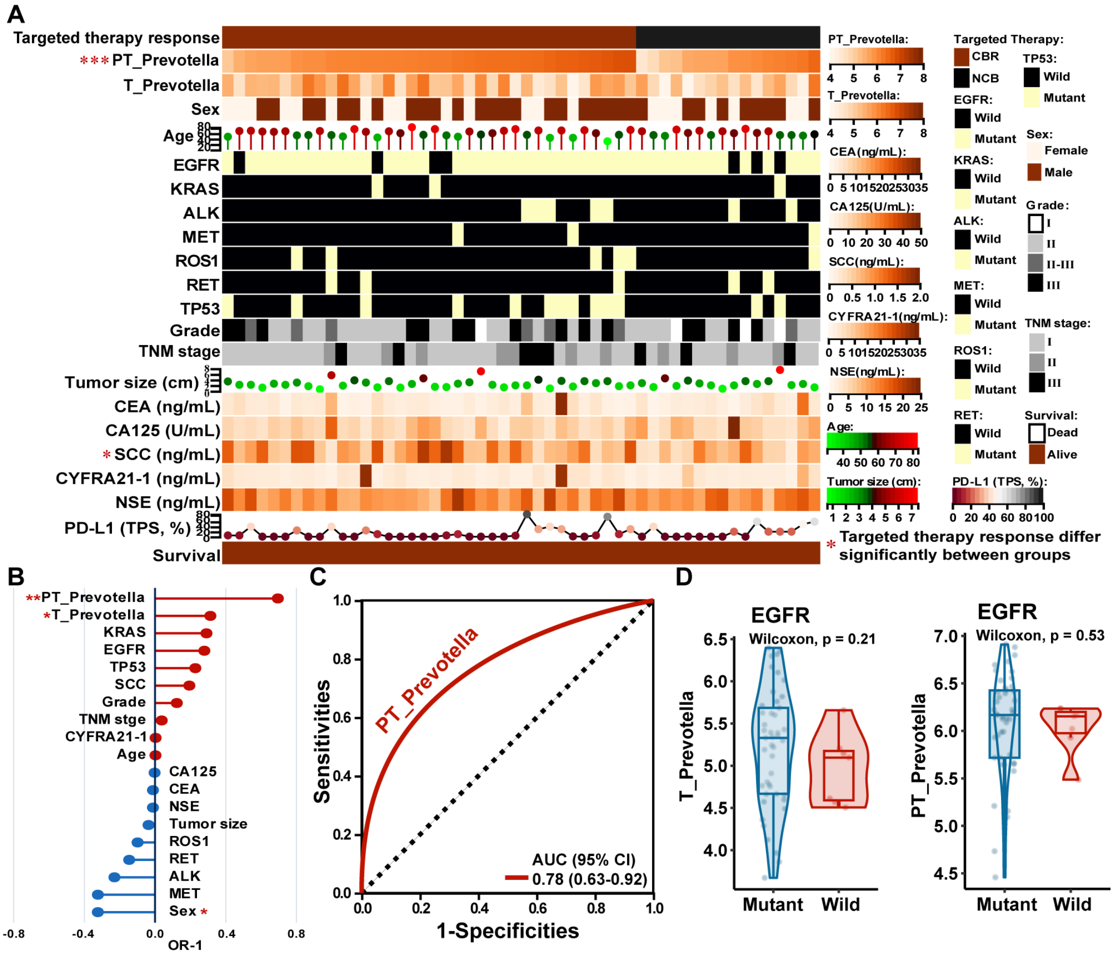

A
Targeted therapy response
***PT_Prevotella
T_Prevotella
Sex
Age
EGFR
KRAS
ALK
MET
ROS1
RET
TP53
Grade
TNM stage
Tumor size (cm)
CEA (ng/mL)
CA125 (U/mL)
*SCC (ng/mL)
CYFRA21-1 (ng/mL)
NSE (ng/mL)
PD-L1 (TPS, %)
Survival
PT_Prevotella:
T_Prevotella:
CEA(ng/mL):
CA125(U/mL):
SCC(ng/mL):
CYFRA21-1(ng/mL):
NSE(ng/mL):
Age:
Tumor size (cm):
Targeted Therapy:
CBR
NCB
TP53:
EGFR:
KRAS:
ALK:
MET:
ROS1:
RET:
Wild
Mutant
Sex:
Female
Male
Grade:
I
II
II-III
III
TNM stage:
Survival:
Dead
Alive
PD-L1 (TPS, %):
* Targeted therapy response differ significantly between groups
B
**PT_Prevotella
*T_Prevotella
KRAS
EGFR
TP53
SCC
Grade
TNM stge
CYFRA21-1
Age
CA125
CEA
NSE
Tumor size
ROS1
RET
ALK
MET
Sex *
OR-1
C
Sensitivities
1-Specificities
PT_Prevotella
AUC (95% CI)
0.78 (0.63-0.92)
D
EGFR
Wilcoxon, p = 0.21
T_Prevotella
Mutant
Wild
EGFR
Wilcoxon, p = 0.53
PT_Prevotella
Mutant
Wild

Figure S5

Figure legends

Figure S1. Analysis of the composition of bacterial microbiota in LUAD and LUSC tissues. (**A**) Composition features of the microbiota in LUAD and LUSC groups at the species level. (**B**) Abundance of the microbiota in the LUAD and LUSC groups at the species level. (**C**) A Venn diagram exhibited the shared and unique species between the LUAD and LUSC groups. (**D**) Comparison of alpha diversity (Chao1, Shannon index, and Simpson index) between the T and PT groups, Statistical significance was determined by two-sided Wilcoxon rank-sum test, *$P < 0.05$. (**E**) Comparison of β-diversity between the tumor (T) and paracancerous tissue (PT) groups based on the Binary Jaccard distance. Statistical significance was assessed using PERMANOVA performed with the ADONIS function, $p < 0.001$.

Figure S2. Differential abundances of bacterial taxa between the LUAD and LUSC groups. (**A**, **B**) Boxplots showed that three geneus (**A**) and five species (**B**) among the top ten bacteria in abundance in LUAD and LUSC differed between groups as determined by two-sided paired Wilcoxon signed-rank tests, $P < 0.05$ considered statistically significant. (**C**) Cladogram generated by the LEfSe represents the taxonomic hierarchical structure of the identified microbial populations. Red nodes and green nodes represent relatively high abundance of species with significant difference in LUAD and LUSC group, respectively. Yellow nodes indicate that there was no significant difference in the comparison of species in the two groups. (**D**) The histogram of LDA score showed 7 biomarkers with significant differences between the LUAD and LUSC group. LDA score represented the influencing degree of biomarkers.

Figure S3. Screening of LUAD and LUSC, LUADP and LUSCP gene-dependent microbes based on CMI technology. (**A**) Microbes-host interaction network in T group constructed using CMI technology. (**B**) 2 of the 24 gene-dependent genera in the microbes-host interaction network differed between the LUAD and LUSC groups. (**C**) Microbes-host interaction network in PT group constructed using CMI technology. (**D**) No genus in the 24 gene-dependent genera in the microbes-host interaction network differed between the LUADP and LUSCP groups.

Figure S4. Immunohistochemical (IHC) results showing the consistency between the abundance of paracancerous tissue-resident *Prevotella* and PD-L1 expression in tumor tissues.

Figure S5. Effect of paracancerous tissue (PT)-resident *Prevotella* on target therapy response of NSCLC. (**A**) The heatmap displays the distribution of tissue-resident *Prevotella* and clinical indicators between the CBR and NCB groups in the target therapy cohort. (**B**) The Generalized Linear Mixed Model (GLMM) identifies independent indicators associated with target therapy response. (**C**) The ROC curve for predicting immunotherapy response using the PT-resident *Prevotella*. (**D**) The difference in the abundance of tumor and PT-resident *Prevotella* between the *EGFR* mutation and wild-type groups
